# On the Use of Cinchona in Ophthalmia

**Published:** 1810-09

**Authors:** W. Hamilton

**Affiliations:** Ipswich


### *To the Editors of the Medical and Phyſical Journal.*

GENTLEMEN,

ON perusing the last Number of your useful Miscellany, my attention was drawn to Mr. Headley's Cases and Observations on Ophthalmia. The purport of this paper seems to be to call the attention of medical men to the administration nation of the bark in the intermittent form of this disease. The hint of the use of this remedy, in such cases, he candidly confesses, he received from the late Mr. Saunders.

As the tenour of this communication might lead some to suppose, that the efficacy of this bark in ophthalmy was not generally known, I beg leave to observe to Mr. H. that it has been used for many years in that intermittent form of the disease to which he alludes. To prove this assertion, it is only necessary to refer to the works of that celebrated oculist Mr. Ware. Its use is first mentioned in his Observations on Scrophulous and Intermittent Ophthalmy, published in 1792, and afterwards in the first volume of his Chirurgical Observations, relative to the eye, &c. promulgated in 1805.

Here, however, he has judged it necessary to caution practitioners against its indiscriminate use, adding, "I feel it my duty to caution against the hasty adoption of this remedy, since the instances in which it is likely to prove useful are extremely rare, and if it be improperly employed in such large doses, it will increase the inflammation and much aggravate all the symptoms." Notwithstanding this, it appears to have been useful in several of the cases which Mr. Ware relates. He, however, gives the preference, in the intermittent form of the disease, to a solution of oxymuriate of mercury taken inwardly, in about the proportion of a quarter of a grain every night in any proper vehicle. Of the good effects of this remedy he thus expresses himself, "I have the satisfaction to add, that in a great variety of such cases, I have afforded the most striking relief by means of it, after the cortex peruvianus and various other medicines had been found totally incompetent for this purpose."

I may add, that I have had an opportunity of using it in two instances, in which its application, both internally and externally, speedily produced a cure, after the common means were tried in vain.

As to the cinchona, there is no doubt but it will be found useful as a powerful tonic, in many of these chronic, debilitated, and intermittent forms which the disease often assumes.

If I have used any expression in these cursory remarks, which may in the least tend to hurt Mr. H's feelings, I ask pardon, and beg leave to assure him, that the diffusion of useful knowledge, and not criticism, was my aim.

I am, &c.

W. HAMILTON.

